# Risk of Ischemic Bowel Disease in Patients With Atrial Fibrillation Receiving Warfarin or Non-vitamin K Antagonist Oral Anticoagulants

**DOI:** 10.3389/fcvm.2022.874460

**Published:** 2022-07-05

**Authors:** Jo-Nan Liao, Yi-Hsin Chan, Ling Kuo, Chuan-Tsai Tsai, Su-Shen Lim, Tze-Fan Chao

**Affiliations:** ^1^Division of Cardiology, Department of Medicine, Taipei Veterans General Hospital, Taipei, Taiwan; ^2^Institute of Clinical Medicine, and Cardiovascular Research Center, National Yang Ming Chiao Tung University, Taipei, Taiwan; ^3^The Cardiovascular Department, Chang Gung Memorial Hospital, Taoyuan, Taiwan; ^4^College of Medicine, Chang Gung University, Taoyuan, Taiwan; ^5^Microscopy Core Laboratory, Chang Gung Memorial Hospital, Taoyuan, Taiwan

**Keywords:** atrial fibrillation, ischemic bowel disease, anticoagulant, NOACs, warfarin

## Abstract

**Background:**

Although atrial fibrillation (AF) is a risk factor for ischemic bowel disease, data regarding the incidence of ischemic bowel disease in patients with anticoagulated AF were limited.

**Methods:**

The present study used the Taiwan NHIRD and included newly diagnosed patients with AF aged ≥ 20 years without ischemic bowel disease from 2012 to 2018. A total of 69,549 patients taking warfarin or non-vitamin K antagonist oral anticoagulants (NOACs) constituted the final study group. We aimed to study the incidence of ischemic bowel disease in patients with AF receiving warfarin or NOACs. Secondary endpoints were also analyzed, including ischemic stroke, systemic embolism, myocardial infarction, mortality, intracranial hemorrhage (ICH), major bleeding, and composite adverse events (ischemic bowel disease or ICH or major bleeding).

**Results:**

There were 43,787 patients taking NOACs and 25,762 patients taking warfarin. The overall incidence rate of ischemic bowel disease was 0.036% per year and increased with the CHA_2_DS_2_-VASc scores [0.013% for patients with a CHA_2_DS_2_-VASc score of 0 (men) or 1 (women), 0.022% for those with a CHA_2_DS_2_-VASc score of 1 (men) or 2 (women), and 0.039% for those with a CHA_2_DS_2_-VASc score ≥ 2 (men) or ≥ 3 (women)]. The risk of ischemic bowel disease was similar between NOAC and warfarin groups (0.036%/year vs. 0.037%/year; adjusted hazard ratio 0.802, *p* = 0.430), whereas the NOAC group had a significantly lower risk of secondary endpoints compared to the warfarin group.

**Conclusion:**

We reported the incidence of ischemic bowel disease in patients with anticoagulated AF from a nationwide cohort database and observed a positive correlation between the increase of CHA_2_DS_2_-VASc scores and the incidence rate. Moreover, NOAC was as effective as warfarin for the risk of ischemic bowel disease.

## Introduction

Ischemic bowel disease encompasses a heterogeneous group of disorders characteristic of inadequate oxygenated blood supply to any part of the bowel walls (1) and associated with high morbidity and mortality rates. Ischemic bowel disease includes acute and chronic mesenteric ischemia affecting the small bowel and colon ischemia (1, 2) with the main pathophysiologic process of embolism, arterial or venous thrombosis, and non-occlusive mesenteric ischemia (3, 4). Atherosclerosis, hypoperfusion as a result of arrhythmia, heart failure, or shock, hypercoagulable state, vasculitis, or any mechanical obstruction to the mesenteric blood vessels may also lead to ischemic bowel disease (1). Embolism to the visceral vessels is the most common cause of mesenteric ischemia, responsible for approximately 30–50% of cases, and is often seen in the context of atrial fibrillation (AF), structural heart disease, or ischemic heart disease (4–6).

AF increases the risk of ischemic stroke and systemic embolism (IS/SE) (7), and oral anticoagulants (OACs) are recommended in high-risk patients unless contraindications exist (8, 9). Because of comparable or better efficacy and better safety, non-vitamin K antagonist oral anticoagulants (NOACs) have replaced warfarin as the mainstream OAC for stroke prevention. AF as a risk factor for ischemic bowel disease is justified by the tendency of embolism. Besides, AF-related irregular heart rhythm may predispose to hypoperfusion and ischemia of the bowel walls (1). Although the relationship between AF and ischemic bowel disease is legitimate and recognized, data regarding incident ischemic bowel disease in patients with anticoagulated AF remain limited. Therefore, we aimed to analyze and compare the incidence of ischemic bowel disease in patients with AF receiving warfarin or NOACs, which was not reported in the prior randomized trials.

## Materials and Methods

We used the “National Health Insurance Research Database (NHIRD)” provided by the Health and Welfare Data Science Center (HWDC), Ministry of Health and Welfare (MOHW), Taiwan in the present study. The National Health Insurance (NHI) system is a mandatory universal health insurance program that covers comprehensive medical care for all Taiwanese residents. The NHIRD consists of detailed health care data of > 23 million enrollees, representing > 99% of Taiwan’s population. Patients’ original identification numbers have been encrypted in this cohort dataset to protect privacy, but the encrypting procedure was consistent so that a linkage of the claims belonging to the same patient was feasible within the NHI database and can be followed continuously. Details about Taiwan NHIRD have been provided in our previous studies (10–20), and the accuracy of the NHIRD has been validated (21, 22).

### Study Population

**FIGURE 1:**
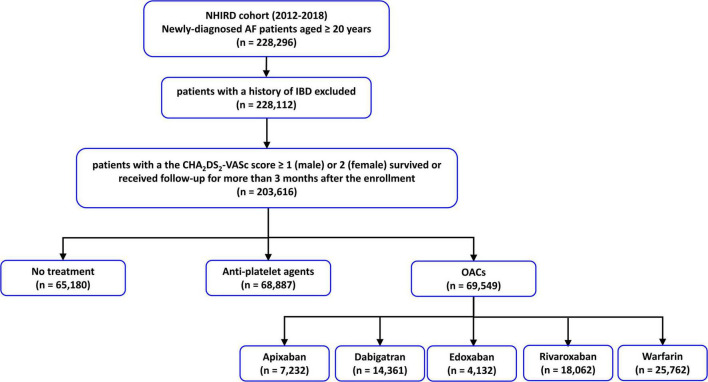
The flowchart of the enrollment of study patients. AF, atrial fibrillation; IBD, ischemic bowel disease; NHIRD, National Health Insurance Research Database; NOACs, non-vitamin K antagonist oral anticoagulants; OACs, oral anticoagulants.

From 1 January 2012 to 31 December 2018, 228,112 patients aged ≥ 20 years newly diagnosed with AF without a history of ischemic bowel disease were identified from NHIRD. The diagnosis of AF was identified by using the International Classification of Diseases, Ninth Revision (ICD-9), Clinical Modification codes (427.31), which were registered by the responsible physicians. The diagnosis of ischemic bowel disease was based on the ICD-9-CM codes 557.0, 557.1, and 557.9. The diagnostic accuracy of AF and ischemic bowel disease using this definition in NHIRD has been validated before (23–25). Among these patients, 203,616 of them had a CHA_2_DS_2_-VASc score ≥ 1 (men) or ≥ 2 (women), and 69,549 patients receiving OACs (25,762 with warfarin, 7,232 with apixaban, 14,361 with dabigatran, 4,132 with edoxaban, and 18,062 with rivaroxaban) have constituted the study population. The flowchart of patient enrollment and study design is shown in Figure 1.

### The CHA_2_DS_2_-VASc, HAS-BLED Scores, and Clinical Endpoints

The CHA_2_DS_2_-VASc score was calculated for each patient by assigning 1 point each for the history of hypertension, diabetes, heart failure, vascular disease (myocardial infarction or peripheral artery disease), female sex, and age between 65 and 74 years and 2 points each for a history of ischemic stroke or transient ischemic attack (TIA) or age ≥ 75 years (26). The HAS-BLED score was calculated by assigning 1 point each for the history of hypertension, abnormal renal or liver function, stroke, bleeding history, age ≥ 65 years, and antiplatelet drug or alcohol use (27). Abnormal renal or liver function was defined by the ICD-9-CM codes rather than laboratory data. The component of “labile INR (international normalized ratio)” was excluded from the scoring scheme in the present study because the INR of warfarin was not available in the registry database. The primary endpoint was the occurrence of ischemic bowel disease. Secondary endpoints included systemic embolism, myocardial infarction, mortality, ICH, major bleeding, and composite adverse events (ischemic bowel disease or ICH or major bleeding).

### Statistical Analysis

Continuous variables were expressed as mean and categorical variables as standard deviation and proportions. Differences between continuous values were assessed using the unpaired two-tailed *t*-test, and differences between nominal variables were compared by the chi-squared test. The incidence rates of events were calculated by dividing the number of events across the entire study period by person-year at risk. The risk of clinical events between NOAC and warfarin was compared using multivariate Cox regression analysis. The risk of events was assessed using Cox regression analysis adjusted for age, sex, and variables which were significantly different between patients receiving NOACs and warfarin. Since multiple clinical factors have already been incorporated into the CHA_2_DS_2_-VASc and HAS-BLED scores, which were used to represent the stroke and bleeding risk, respectively, only the comorbidity which was not included in these scoring schemes and significantly differed between the 2 groups was adjusted for, in the multivariable Cox regression model. To overcome the differences in baseline demographics, propensity matching analysis was also done between the warfarin and NOAC groups. All statistical significances were set at a *p*-value < 0.05.

## Results

Baseline characteristics of patients are shown in Table 1. There were marked differences in baseline demographic and underlying diseases between the NOAC and warfarin groups. Patients taking NOACs were older, had more comobidities of hypertension, diabetes mellitus, stroke, vascular disease, chronic obstructive pulmonary disease, hyperlipidemia, autoimmune diseases, cancer, hyperthyroidism, abnormal liver function, history of bleeding, and had less congestive heart failure, abnormal renal function, anemia, alcohol excess/abuse, and gout. In general, the NOAC group had higher CHA_2_DS_2_-VASc and HAS-BLED scores than the warfarin group.

**TABLE 1:** Baseline characteristics of NOAC and warfarin groups.

Variables	NOACs *N* = 43,787	Warfarin *N* = 25,762	*p*
Age, years; mean value (SD)	75.73 (10.69)	70.06 (12.75)	<0.001
Age ≥ 75 years, *n* (%)	25,734 (58.77)	10,321 (40.06)	<0.001
Age 65–74 years, *n* (%)	12,093 (27.62)	6,811 (26.44)	<0.001
Male sex, *n* (%)	23,699 (54.12)	14,286 (55.45)	<0.001
CHA_2_DS_2_-VASc score; mean values (SD)	4.20 (1.75)	3.68 (1.96)	<0.001
HAS-BLED score, mean value (SD)	2.89 (1.27)	2.61 (1.43)	<0.001
**Comorbidities, *n* (%)**			
Hypertension	36,542 (83.45)	19,337 (75.06)	<0.001
Diabetes mellitus	16,792 (38.35)	8,815 (34.22)	<0.001
Congestive heart failure	16,532 (37.76)	11,299 (43.86)	<0.001
Stroke	12,587 (28.75)	6,838 (26.54)	<0.001
Vascular diseases	5,112 (11.67)	2,756 (10.7)	<0.001
COPD	11,232 (25.65)	5,784 (22.45)	<0.001
Hyperlipidemia	23,545 (53.77)	11,748 (45.6)	<0.001
Autoimmune diseases	2,995 (6.84)	1,489 (5.78)	<0.001
Cancer	5,610 (12.81)	2,631 (10.21)	<0.001
Hyperthyroidism	879 (2.01)	459 (1.78)	0.036
Abnormal renal function	7,911 (18.07)	5,227 (20.29)	<0.001
Abnormal liver function	9,144 (20.88)	4,783 (18.57)	<0.001
Anemia	5,068 (11.57)	3,686 (14.31)	<0.001
History of bleeding	11,923 (27.23)	6,702 (26.02)	<0.001
Alcohol excess/abuse	584 (1.33)	414 (1.61)	0.003
Gout	8,984 (20.52)	5,635 (21.87)	<0.001

*COPD, chronic obstructive pulmonary disease; NOACs, non-vitamin K antagonist oral anticoagulants; SD, standard deviation.*

### Incidence and Risk of Ischemic Bowel Disease

**FIGURE 2:**
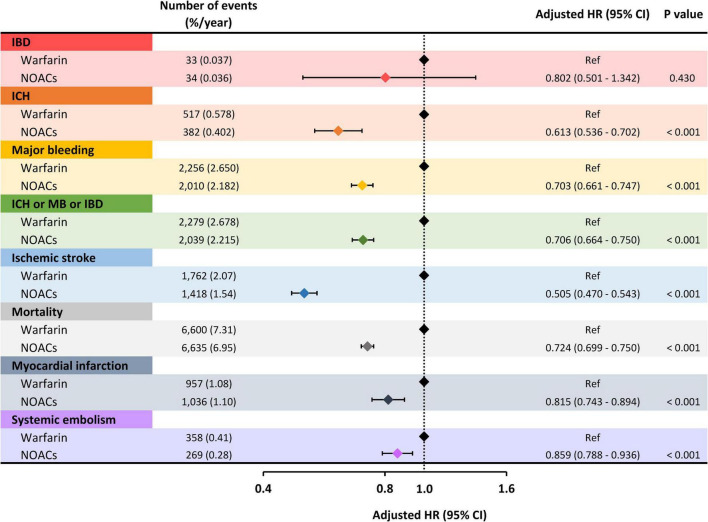
Risk of clinical events of NOACs compared to warfarin. The risk of ischemic bowel disease was similar between the NOAC and warfarin groups. As for secondary endpoints, including ICH, major bleeding, ischemic stroke, mortality, myocardial infarction, and systemic embolism, NOACs were associated with a significantly lower risk compared to warfarin. HR, hazard ratio; CI, confidence interval; IBD, ischemic bowel disease; ICH, intracranial hemorrhage; MB, major bleeding; NOACs, non-vitamin K antagonist oral anticoagulants.

During the follow-up period, 67 patients experienced ischemic bowel disease with an annual incidence of 0.036% (Table 2). The incidence rates of ischemic bowel disease were proportional to the increase of the CHA_2_DS_2_-VASc score as follows: 0.013% for patients with a CHA_2_DS_2_-VASc score of 0 (men) or 1 (women), 0.022% for those with a CHA_2_DS_2_-VASc score of 1 (men) or 2 (women), and 0.039% for those with a CHA_2_DS_2_-VASc score ≥ 2 (men) or ≥ 3 (women) (Table 2). Multivariate Cox regression analysis adjusted for age, sex, CHA_2_DS_2_-VASc score, HAS-BLED score, chronic obstructive pulmonary disease, hyperlipidemia, autoimmune diseases, cancer, hyperthyroidism, and gout observed a similar risk of ischemic bowel disease between the NOAC and warfarin groups [adjusted hazard ratio (aHR): 0.802; 95% confidence interval (CI): 0.501–1.342; *p* = 0.430] (Figure 2). We also performed a propensity score matching analysis that showed the same results between the NOACs and warfarin groups (HR: 0.886; 95% CI: 0.422–1.775; *p* = 0.598).

**TABLE 2:** Incidence of IBD in relation to the CHA_2_DS_2_-VASc score.

CHA_2_DS_2_-VASc score	*n*	Event number of IBD	Person-year	Incidence rate (% per year)
All	69,549	67	185,659	0.036
0 (male) or 1 (female)	2,470	1	7,831	0.013
1 (male) or 2 (female)	6,046	4	18,021	0.022
≥2 (male) or ≥3 (female)	61,033	62	159,807	0.039

*IBD, ischemic bowel disease.*

### Risks of Secondary Endpoints in Patients Receiving Non-vitamin K Antagonist Oral Anticoagulants or Warfarin

Compared to warfarin use, Non-vitamin K Antagonist Oral Anticoagulant (NOAC) was associated with a lower risk of ICH (aHR: 0.613; 95% CI: 0.536–0.702; *p* < 0.001), major bleeding (aHR: 0.703; 95% CI: 0.661–0.747; *p* < 0.001), composite adverse events (aHR: 0.706; 95% CI: 0.664–0.750; *p* < 0.001), ischemic stroke (aHR: 0.505; 95% CI: 0.470–0.543; *p* < 0.001), mortality (aHR: 0.724; 95% CI: 0.699–0.750; *p* < 0.001), myocardial infarction (aHR: 0.815; 95% CI: 0.743–0.894; *p* < 0.001), and systemic embolism (aHR: 0.859; 95% CI: 0.788–0.936; *p* < 0.001) (Figure 2).

### Subgroup Analysis Among Different Non-vitamin K Antagonist Oral Anticoagulants in Relation to the Risk of Ischemic Bowel Disease

**FIGURE 3:**
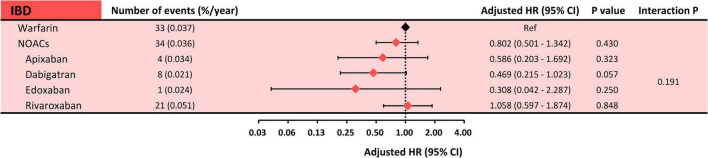
Subgroup analysis: risk of ischemic bowel disease between each NOAC and warfarin. Subgroup analysis showed a comparable risk of ischemic bowel disease between each NOAC and warfarin. The interaction *p*-value was 0.1907. HR, hazard ratio; CI, confidence interval; IBD, ischemic bowel disease; NOACs, non-vitamin K antagonist oral anticoagulants.

Subgroup analysis comparing each Non-vitamin K Antagonist Oral Anticoagulant (NOAC) with warfarin in relation to risks of ischemic bowel disease was done, and the results showed no significant differences between each NOAC and warfarin (apixaban: aHR: 0.586, 95% CI: 0.203–1.692, *p* = 0.3234; dabigatran: aHR: 0.469, 95% CI: 0.215–1.023, *p* = 0.0571; edoxaban: aHR 0.308, 95% CI: 0.042–2.287, *p* = 0.2498; and rivaroxaban: aHR 1.058, 95% CI: 0.597–1.874, *p* = 0.8475). The interaction *p*-value was 0.1901 between the subgroups (Figure 3).

## Discussion

### Main Findings

In this nationwide cohort study, we investigated the risk of ischemic bowel disease in patients with anticoagulated AF and its relationship with the CHA_2_DS_2_-VASc score. The main findings were as follows: (1) the annual incidence of ischemic bowel disease in patients with anticoagulated AF was 0.036% with a positive correlation with the increase of CHA_2_DS_2_-VASc scores and (2) NOACs and warfarin were comparable in the risk of ischemic bowel disease.

### Current Data About the Risk of Ischemic Bowel Disease in Patients With Anticoagulated Atrial Fibrillation

Because of the different entities of ischemic bowel disease, the actual incidence of ischemic bowel disease based on previous literature remains unknown, ranging between 0.09 and 0.2% of acute mesenteric ischemia (1, 28) and 0.16 and 0.18% of colon ischemia (29–31). A retrospective nationwide cohort study by Hu et al. reported an incidence between 0.36 and 1.1%, higher than the results in our study (24). Of note, it is not fair to directly compare the incidence of ischemic bowel disease between different studies because of different study designs and populations. Our study included patients with anticoagulated Atrial Fibrillation (AF) only whereas Hu’s study enrolled patients with AF regardless of OAC use. Besides, clinical management for patients with AF varies in different eras, which might partly explain the difference in reported incidence rates. For example, Hu’s study population was between the years 2000 and 2011, whereas our study population ranged from 2012 to 2018, when OAC use has become more common because of the introduction of NOACs (32, 33). Our report of a lower incidence of 0.036% per year might suggest a beneficial role of anticoagulation in reducing ischemic bowel disease. However, more studies are warranted to support our assumption.

We found that the risk of ischemic bowel disease was proportional to the increase in CHA_2_DS_2_-VASc score, consistent with a previous study that reported a 3.35 times higher risk of ischemic bowel disease in patients with AF with a CHA_2_DS_2_-VASc score ≥ 2 compared to those with a CHA_2_DS_2_-VASc score < 2 (24). However, old age is a well-recognized risk factor of ischemic bowel disease and, at the same time, an important component of the CHA_2_DS_2_-VASc score. Therefore, we cannot exclude the possibility that old age is a principal driver of the increased incidence in patients with AF with high CHA_2_DS_2_-VASc scores. Even so, we observed that, the CHA_2_DS_2_-VASc score, an important risk scheme for ischemic stroke and a must-do in patients with AF, also serves as a useful tool for risk stratification of ischemic bowel disease.

### Bleeding Complications Between the Warfarin and Non-vitamin K Antagonist Oral Anticoagulants Groups

It is important to weigh the benefit against risk when choosing OAC in patients with AF. NOAC is the mainstream OAC for patients with AF because of better or comparable efficacy in preventing ischemic stroke/systemic embolism and less ICH or major bleeding compared to warfarin in randomized controlled trials and real-world cohort studies (32, 34–38). However, to the best of our knowledge, no studies analyzed endpoints of ischemic bowel disease and bleeding under different OAC use in patients with AF at the same time. In the present study, we observed similar effectiveness of NOACs and warfarin in preventing ischemic bowel disease, whereas NOACs were associated with decreased risks of secondary endpoints compared to warfarin. A composite endpoint combining ischemic bowel disease, ICH, and major bleeding was analyzed, and NOACs were associated with a 29% decrease in risk compared to warfarin. Our results imply that NOAC might be a better choice than warfarin balancing effectiveness and the risk of bleeding.

### Limitations

Our study had some limitations. First, the present nationwide cohort study was based on the Taiwan National Insurance database and may be limited by the coding system that does not completely reflect types of ischemic bowel disease. Therefore, details of ischemic bowel disease were unknown, and the types of ischemic bowel disease with pathologic processes other than embolism also should have been included in the analysis. However, atherosclerosis is also an important mechanism underlying AF-associated adverse consequences and might be involved in ischemic bowel disease. Although our findings cannot represent a true causal relationship between AF and ischemic bowel disease, we demonstrated that these two are closely intertwined and increased the risk of ischemic bowel disease with the increase of the CHA_2_DS_2_-VASc scores. Second, ischemic bowel disease is sometimes overlooked in clinical practice, especially in elderly patients, besides the possibility of underdiagnosis/under-coding of ischemic bowel disease in the retrospective administrative databases, which might be one of the reasons underlying a low incidence of ischemic bowel disease in our report. Nevertheless, what we tried to report is the incidence of ischemic bowel disease in patients with anticoagulated AF, which has not been well studied before. Besides, underdiagnosis/underreporting can be present in both warfarin and NOAC groups and may not interfere with the focus of our study. Third, although we observed a similar risk of incident ischemic bowel disease between the NOACs and warfarin groups, the analysis may be limited by the low event number. Fourth, how types of AF influence the risk of ischemic bowel disease is unknown since types of AF were unavailable in the database. However, current guidelines suggested OAC use in AF patients with a high risk of systemic embolism irrespective of the type of AF, so the lack of AF types in the database may not influence the decision of OAC. Fifth, detailed information about echocardiographic parameters was unavailable in the database. However, this limitation is present in both warfarin and NOAC groups and the choice of OAC should not be influenced by echocardiographic parameters. Last, marked differences in baseline characteristics and underlying diseases are common in real-world cohort databases. Although we used the multivariate Cox regression analysis and the propensity score matching analysis to overcome the baseline differences, potential confounding factors cannot be excluded.

### Clinical Implication and Future Directions

Despite current advances in diagnostic tools for most gastrointestinal diseases, ischemic bowel disease remains a highly morbid disease relying on a high degree of clinical suspicion and prompt management (6). A focused review of underlying history along with clinical symptoms/signs is crucial to improve the diagnostic rate. Since patients with AF are susceptible to an increased risk of ischemic bowel disease and prone to the complicated clinical course, a high awareness is important for timely detection. The CHA_2_DS_2_-VASc score is the most used scheme for the risk of ischemic stroke in patients with AF, and we proved that it can also be applied for risk stratification of ischemic bowel disease.

Although inadequate anticoagulation is associated with mesenteric ischemia in patients with AF (39), data regarding incidence rates of ischemic bowel disease in patients with AF are still limited, not to mention the scarcity of evidence in patients with anticoagulated AF. Besides, previous studies mostly used warfarin as the main OAC, whereas NOACs have largely replaced warfarin nowadays. In the present study, we used a nationwide cohort database to report the incidence of ischemic bowel disease in patients with anticoagulated AF and compared the incidence of various adverse events, especially ischemic bowel disease, between patients with AF taking warfarin and NOACs.

Despite all the efforts trying to improve the accuracy and minimize baseline differences, potential biases cannot be excluded. Future studies are needed to identify the risk of ischemic bowel disease between patients with AF and non-AF patients and to prove the beneficial effect of OAC in AF-related ischemic bowel disease. For example, prospective studies including detailed information on ischemic bowel disease in relation to treatment strategies should be done since a relatively low incidence of ischemic bowel disease in the present study may fail to tell prognostic differences between warfarin and NOACs. Furthermore, studies comparing the use of antiplatelet agents and OACs might be helpful to clarify the effect of OAC for ischemic bowel disease in patients with AF.

## Conclusion

This nationwide cohort study showed that the incidence of ischemic bowel disease was around 0.036%/year in patients with anticoagulated AF and proportional to the increase of the CHA_2_DS_2_-VASc scores. NOACs showed similar effectiveness to warfarin for the risk of ischemic bowel disease.

## Data Availability Statement

The original contributions presented in this study are included in the article/supplementary material, further inquiries can be directed to the corresponding author/s.

## Ethics Statement

The studies involving human participants were reviewed and approved by the Institutional Review Board, Taipei Veterans General Hospital. Written informed consent for participation was not required for this study in accordance with the national legislation and the institutional requirements.

## Author Contributions

J-NL was responsible for manuscript writing. Y-HC was responsible for database resources. LK was responsible for data analysis. C-TT was responsible for idea conceptualization. S-SL was responsible for editing figures. T-FC was responsible for key ideas and organization. All authors contributed to the article and approved the submitted version.

## Conflict of Interest

The authors declare that the research was conducted in the absence of any commercial or financial relationships that could be construed as a potential conflict of interest.

## Publisher’s Note

All claims expressed in this article are solely those of the authors and do not necessarily represent those of their affiliated organizations, or those of the publisher, the editors and the reviewers. Any product that may be evaluated in this article, or claim that may be made by its manufacturer, is not guaranteed or endorsed by the publisher.
